# 5-(4-Chloro­phen­yl)-3-(4-meth­oxy­phen­yl)iso­thia­zole

**DOI:** 10.1107/S2414314626010989

**Published:** 2026-10-30

**Authors:** A. S. Jeevan Chakravarthy, H. D. Kavitha, N. R. Sreenatha

**Affiliations:** a Department of Chemistry BMS Institute of Technology & Management Autonomous under Visvesvaraya Technological University, Avalahalli Yelahanka Bengaluru 560064 Karnataka India; b Department of Physics, Government First Grade College, Pandavapura, Mandya, Karnataka 571434, India; c Research Center, Department of Physics, Government Engineering College, Bedarapura, Chamarajanagara 571313, Karnataka, India; University of Aberdeen, United Kingdom

**Keywords:** chloro­phen­yl, meth­oxy­phen­yl, iso­thia­zole, C—H⋯N/S interactions, crystal structure

## Abstract

In the title compound, C_16_H_12_ClNOS, the dihedral angles between the central iso­thia­zole ring and pendant *p*-meth­oxy­phenyl and *p*-chloro­phenyl rings are 19.30 (19) and 13.8 (2)°, respectively. The mol­ecule exhibits short intra­molecular C—H⋯S and C—H⋯N contacts, each generating an *S*(5) ring motif.

## Abstract 2

In the title compound, the dihedral angles between the central iso­thia­zole ring and pendant *p*-meth­oxy­phenyl and *p*-chloro­phenyl rings are 19.30 (19) and 13.8 (2)°, respectively. The mol­ecule exhibits short intra­molecular C—H⋯S and C—H⋯N inter­actions, each generating an *S*(5) ring motif.

## Graphical abstract

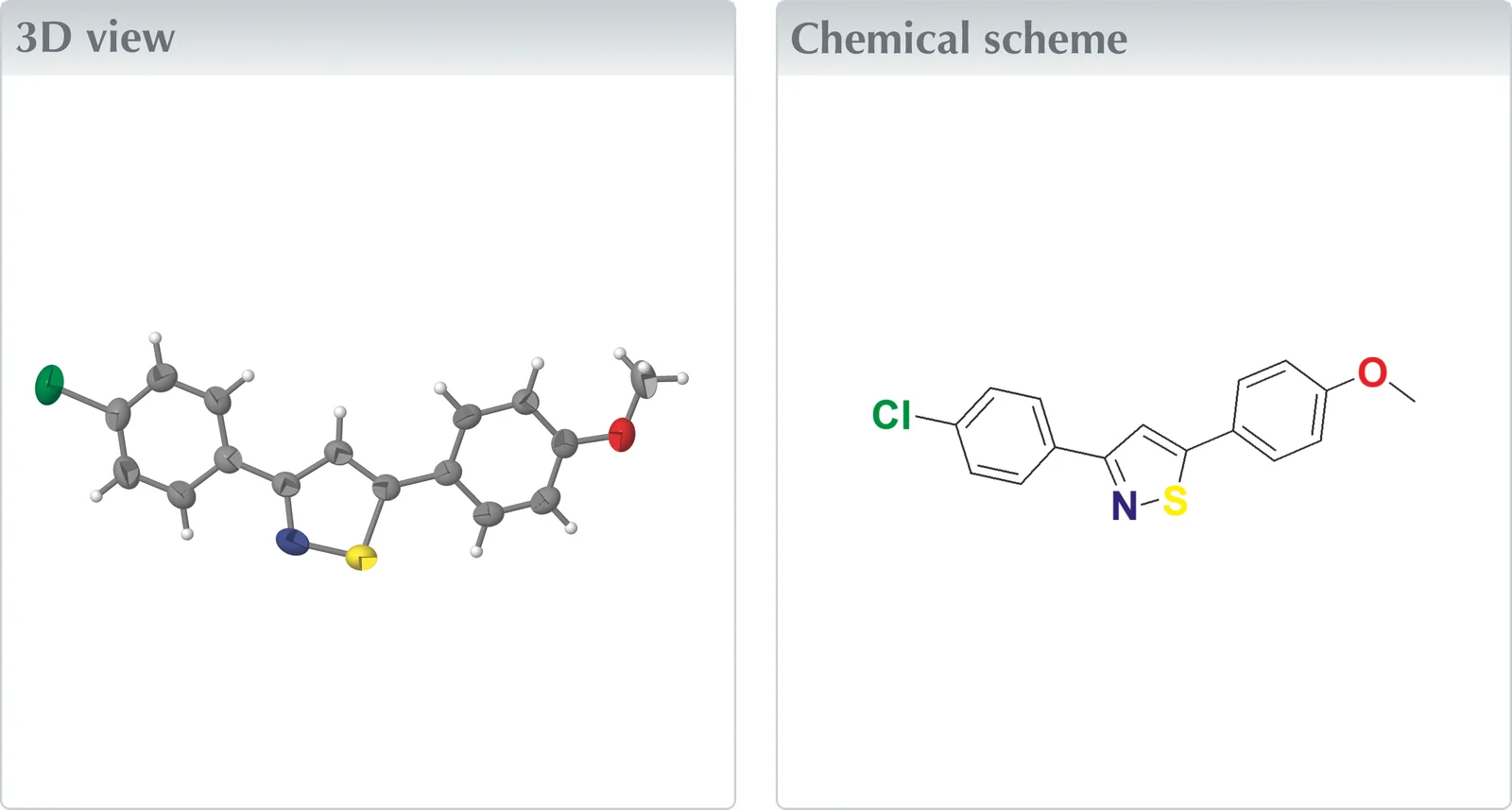

## Structure description

**Figure 1:**
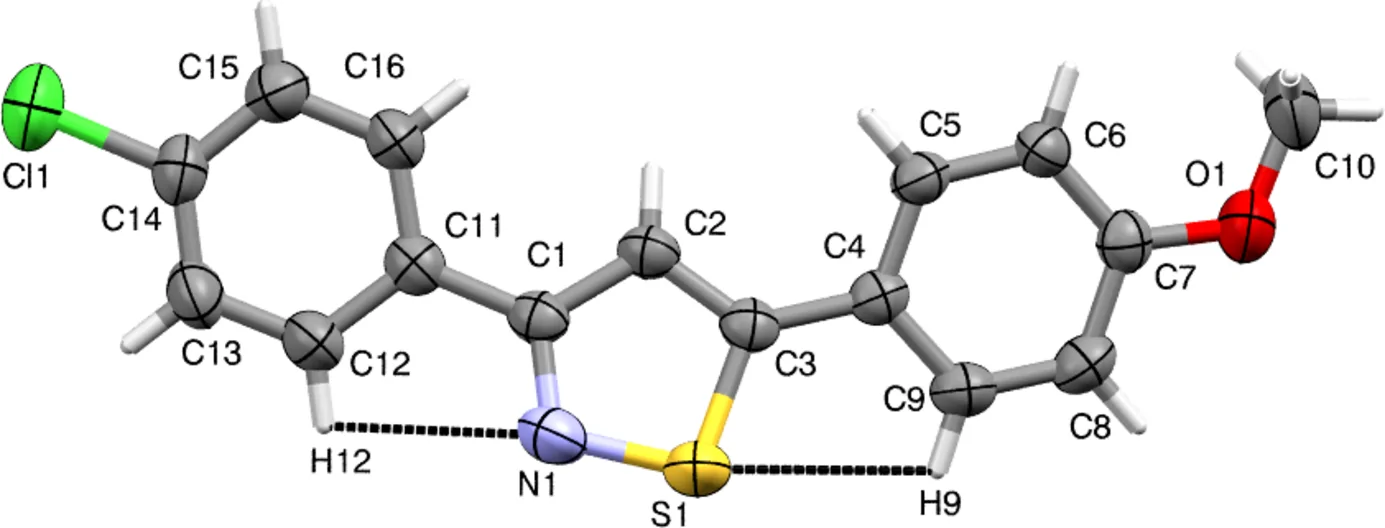
The mol­ecular structure of **2** drawn at 50% probability with short intra­molecular contacts represented by dashed lines.

**Figure 2:**
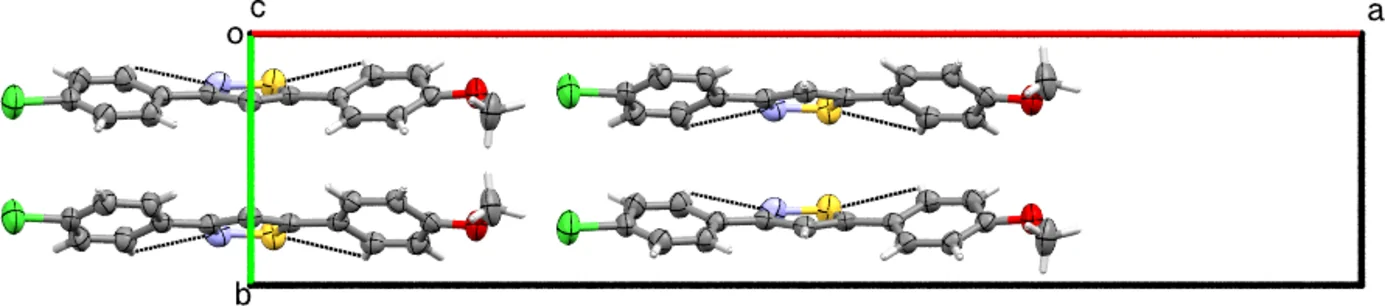
The packing for **2** viewed along the crystallographic *c* axis.

The title compound, C_16_H_12_ClNOS (**2**), crystallizes with one mol­ecule in the asymmetric unit (Fig. 1 ▸) in space group *Cc*. The dihedral angles between the central C1/C2/C3/S1/N1 iso­thia­zole ring and pendant C4–C9 *p*-meth­oxy­phenyl and C11–C16 *p*-chloro­phenyl rings are 19.30 (19) and 13.8 (2)°, respectively. The dihedral angle between the pendant rings is 15.9 (2)°. The inter-ring bond distances of C1—C11 = 1.472 (6) Å and C3—C4 = 1.461 (5) Å are very similar. Otherwise, all the bond distances in **2** are consistent with those in related structures (Chakravarthy *et al.*, 2026 ▸; Sreenatha *et al.*, 2020 ▸; Sreenatha *et al.*, 2018 ▸). The mol­ecular conformation of **2** is supported by short intra­molecular C9—H9⋯S1 and C12—H12⋯N1 contacts (Table 1 ▸): both of these enclose an *S*(5) ring. The packing of the mol­ecules is shown in Fig. 2 ▸.

## Synthesis and crystallization

**Figure 3:**
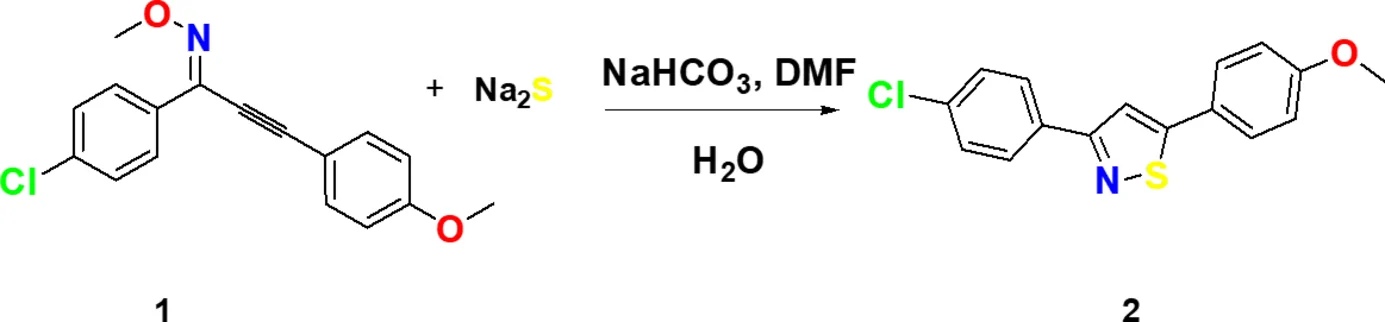
Synthesis scheme for **2**.

Compound **2** was synthesized by following a literature procedure (Zhang *et al.*, 2021 ▸) (Fig. 3 ▸). A vessel was charged with **1** (0.20 mmol), Na_2_S (0.30 mmol, 23.4 mg), NaHCO_3_ (0.40 mmol, 33.6 mg) and H_2_O (1.20 mmol, 21.6 mg) in *N*,*N*-di­methyl­formamide (DMF) (2 ml). The reaction mixture was stirred at 90°C in an oil bath for 13–15 h until the starting material was consumed completely as monitored by TLC. After the completion of the reaction the mixture was poured into ethyl acetate and washed with brine (2 × 15 ml). The combined organic layers were dried over anhydrous MgSO_4_ and evaporated under vacuum. The residue was purified by flash column chromatography using petroleum ether/ethyl acetate as the eluent to afford the desired product **2** in 87% yield. Then, 0.5 g of **2** was dissolved in 5 ml of DMF and was allowed to undergo slow evaporation to form colorless blocks.

## Refinement

Crystal data and structure refinement details are summarized in Table 2 ▸.

## Supplementary Material

Crystal structure: contains datablock(s) I. DOI: 10.1107/S2414314626010989/hb4577sup1.cif

Structure factors: contains datablock(s) I. DOI: 10.1107/S2414314626010989/hb4577Isup2.hkl

Supporting information file. DOI: 10.1107/S2414314626010989/hb4577Isup3.cml

CCDC reference: 2158969

Additional supporting information:  crystallographic information; 3D view; checkCIF report

## Figures and Tables

**Table 1:** Hydrogen-bond geometry (Å, °)

*D*—H⋯*A*	*D*—H	H⋯*A*	*D*⋯*A*	*D*—H⋯*A*
C9—H9⋯S1	0.93	2.77	3.155 (4)	106
C12—H12⋯N1	0.93	2.55	2.857 (6)	100

**Table 2:** Experimental details

Crystal data	
Chemical formula	C_16_H_12_ClNOS
*M*_r_	301.78
Crystal system, space group	Monoclinic, *Cc*
Temperature (K)	301
*a*, *b*, *c* (Å)	32.054 (3), 7.2423 (7), 6.0942 (5)
β (°)	93.607 (3)
*V* (Å^3^)	1411.9 (2)
*Z*	4
Radiation type	Mo *K*α
μ (mm^−1^)	0.41
Crystal size (mm)	0.30 × 0.27 × 0.21

Data collection	
Diffractometer	Bruker APEXII
Absorption correction	Multi-scan (Krause *et al.*, 2015 ▸)
*T*_min_, *T*_max_	0.677, 0.739
No. of measured, independent and observed [*I* > 2σ(*I*)] reflections	43343, 4353, 2555
*R*_int_	0.094
(sin θ/λ)_max_ (Å^−1^)	0.715

Refinement	
*R*[*F*^2^ > 2σ(*F*^2^)], *wR*(*F*^2^), *S*	0.048, 0.121, 1.01
No. of reflections	4353
No. of parameters	182
No. of restraints	2
H-atom treatment	H-atom parameters constrained
Δρ_max_, Δρ_min_ (e Å^−3^)	0.21, −0.25
Absolute structure	Flack *x* determined using 978 quotients [(*I*^+^)−(*I*^−^)]/[(*I*^+^)+(*I*^−^)] (Parsons *et al.*, 2013 ▸)
Absolute structure parameter	0.05 (3)

Computer programs: *APEX2* and *SAINT* (Bruker, 2012 ▸), *SHELXT2014/4* (Sheldrick, 2015*a* ▸), *SHELXL2019/2* (Sheldrick, 2015*b* ▸) and *PLATON*(Spek, 2020 ▸).

## full crystallographic data

### 5-(4-Chlorophenyl)-3-(4-methoxyphenyl)-1,2-thiazole Crystal data

**Table:**

C_16_H_12_ClNOS	*F*(000) = 624.0
*M**_r_* = 301.78	*D*_x_ = 1.420 Mg m^−^^3^
Monoclinic, *C**c*	Mo *K*α radiation, λ = 0.71073 Å
*a* = 32.054 (3) Å	Cell parameters from 4353 reflections
*b* = 7.2423 (7) Å	θ = 2.6–30.5°
*c* = 6.0942 (5) Å	µ = 0.41 mm^−^^1^
β = 93.607 (3)°	*T* = 301 K
*V* = 1411.9 (2) Å^3^	Block, colorless
*Z* = 4	0.30 × 0.27 × 0.21 mm

### 5-(4-Chlorophenyl)-3-(4-methoxyphenyl)-1,2-thiazole Data collection

**Table:**

Bruker APEX diffractometer	*R*_int_ = 0.094
Radiation source: graphite	θ_max_ = 30.5°, θ_min_ = 2.6°
*SAINT* (Bruker, 2012) scans	*h* = −45→45
Absorption correction: multi-scan (Krause *et al.*, 2015)	*k* = −10→10
*T*_min_ = 0.677, *T*_max_ = 0.739	*l* = −8→8
43343 measured reflections	4353 standard reflections every 200 reflections
4353 independent reflections	intensity decay: none
2555 reflections with *I* > 2σ(*I*)	

### 5-(4-Chlorophenyl)-3-(4-methoxyphenyl)-1,2-thiazole Refinement

**Table:**

Refinement on *F*^2^	Hydrogen site location: inferred from neighbouring sites
Least-squares matrix: full	H-atom parameters constrained
*R*[*F*^2^ > 2σ(*F*^2^)] = 0.048	*w* = 1/[σ^2^(*F*_o_^2^) + (0.0482*P*)^2^ + 0.4728*P*] where *P* = (*F*_o_^2^ + 2*F*_c_^2^)/3
*wR*(*F*^2^) = 0.121	(Δ/σ)_max_ = 0.018
*S* = 1.01	Δρ_max_ = 0.21 e Å^−^^3^
4353 reflections	Δρ_min_ = −0.25 e Å^−^^3^
182 parameters	Absolute structure: Flack *x* determined using 978 quotients [(*I*^+^)-(*I*^-^)]/[(*I*^+^)+(*I*^-^)] (Parsons *et al.*, 2013)
2 restraints	Absolute structure parameter: 0.05 (3)
Primary atom site location: dual	

### 5-(4-Chlorophenyl)-3-(4-methoxyphenyl)-1,2-thiazole Special details

**Table:**

Geometry. All esds (except the esd in the dihedral angle between two l.s. planes) are estimated using the full covariance matrix. The cell esds are taken into account individually in the estimation of esds in distances, angles and torsion angles; correlations between esds in cell parameters are only used when they are defined by crystal symmetry. An approximate (isotropic) treatment of cell esds is used for estimating esds involving l.s. planes.
Refinement. The methyl H atoms were placed in calculated positions and refined as riding atoms with *U*_iso_(H) = 1.5*U*_eq_(C). All other H atoms were positioned geometrically and refined using a riding model with *U*_iso_(H) = 1.2*U*_eq_(C).

### 5-(4-Chlorophenyl)-3-(4-methoxyphenyl)-1,2-thiazole Fractional atomic coordinates and isotropic or equivalent isotropic displacement parameters (Å^2^)

**Table:**

	*x*	*y*	*z*	*U*_iso_*/*U*_eq_	
N1	0.47015 (14)	0.7064 (5)	0.7742 (6)	0.0591 (11)	
S1	0.52016 (4)	0.70064 (18)	0.83327 (15)	0.0599 (3)	
O1	0.70313 (10)	0.7362 (5)	0.3621 (6)	0.0636 (9)	
Cl1	0.28598 (4)	0.7711 (2)	0.1759 (2)	0.0833 (5)	
C1	0.46277 (13)	0.7425 (5)	0.5613 (7)	0.0400 (9)	
C2	0.49908 (13)	0.7689 (5)	0.4444 (6)	0.0418 (9)	
C3	0.53505 (13)	0.7466 (5)	0.5741 (6)	0.0395 (8)	
C4	0.57864 (12)	0.7502 (5)	0.5173 (6)	0.0382 (8)	
C5	0.59000 (12)	0.8323 (6)	0.3257 (6)	0.0434 (9)	
C6	0.63132 (12)	0.8324 (6)	0.2661 (7)	0.0446 (9)	
C7	0.66182 (13)	0.7482 (6)	0.4015 (7)	0.0433 (9)	
C8	0.65076 (13)	0.6660 (6)	0.5985 (7)	0.0488 (10)	
C9	0.61006 (13)	0.6673 (6)	0.6540 (6)	0.0463 (10)	
C10	0.71611 (17)	0.8111 (10)	0.1694 (10)	0.0847 (18)	
C11	0.41961 (14)	0.7482 (5)	0.4638 (7)	0.0423 (9)	
C12	0.38673 (14)	0.6809 (6)	0.5819 (7)	0.0523 (11)	
C13	0.34575 (14)	0.6873 (6)	0.4948 (8)	0.0586 (12)	
C14	0.33749 (13)	0.7612 (7)	0.2906 (8)	0.0504 (11)	
C15	0.36868 (13)	0.8283 (7)	0.1691 (7)	0.0547 (11)	
C16	0.40983 (13)	0.8195 (6)	0.2572 (7)	0.0492 (10)	
H2	0.498508	0.798179	0.295664	0.050*	
H5	0.569576	0.889094	0.233849	0.052*	
H6	0.638274	0.888710	0.136228	0.054*	
H8	0.671192	0.610591	0.691597	0.059*	
H9	0.603131	0.612082	0.784607	0.056*	
H10A	0.699173	0.763190	0.046890	0.127*	
H10B	0.744828	0.779184	0.153505	0.127*	
H10C	0.713348	0.943035	0.173878	0.127*	
H12	0.392495	0.630994	0.721126	0.063*	
H13	0.324164	0.641881	0.574405	0.070*	
H15	0.362514	0.878733	0.030485	0.066*	
H16	0.431213	0.862731	0.174619	0.059*	

### 5-(4-Chlorophenyl)-3-(4-methoxyphenyl)-1,2-thiazole Atomic displacement parameters (Å^2^)

**Table:**

	*U*^11^	*U*^22^	*U*^33^	*U*^12^	*U*^13^	*U*^23^
N1	0.065 (3)	0.068 (3)	0.045 (2)	−0.0046 (19)	0.0129 (18)	0.0084 (18)
S1	0.0563 (7)	0.0828 (8)	0.0403 (5)	−0.0031 (7)	0.0017 (4)	0.0113 (6)
O1	0.0416 (15)	0.081 (2)	0.068 (2)	0.0024 (16)	0.0012 (14)	0.0022 (19)
Cl1	0.0460 (7)	0.1072 (12)	0.0954 (11)	−0.0022 (7)	−0.0064 (6)	0.0041 (9)
C1	0.047 (2)	0.035 (2)	0.039 (2)	0.0026 (16)	0.0063 (16)	0.0020 (15)
C2	0.048 (2)	0.043 (2)	0.0353 (19)	0.0012 (17)	0.0060 (16)	−0.0001 (15)
C3	0.045 (2)	0.032 (2)	0.041 (2)	−0.0021 (17)	0.0032 (16)	0.0017 (15)
C4	0.045 (2)	0.031 (2)	0.038 (2)	−0.0013 (15)	−0.0020 (15)	−0.0016 (14)
C5	0.046 (2)	0.044 (2)	0.039 (2)	−0.0006 (17)	−0.0050 (17)	0.0050 (17)
C6	0.042 (2)	0.050 (2)	0.041 (2)	−0.0063 (18)	0.0013 (16)	0.0064 (17)
C7	0.041 (2)	0.042 (2)	0.047 (2)	−0.0055 (18)	0.0005 (16)	−0.0048 (17)
C8	0.051 (3)	0.046 (3)	0.048 (2)	0.0055 (18)	−0.0084 (18)	0.0032 (18)
C9	0.055 (2)	0.045 (2)	0.038 (2)	0.0001 (18)	−0.0005 (17)	0.0036 (16)
C10	0.041 (3)	0.130 (5)	0.084 (4)	−0.012 (3)	0.013 (3)	0.006 (4)
C11	0.046 (2)	0.036 (2)	0.045 (2)	0.0000 (17)	0.0069 (17)	−0.0023 (17)
C12	0.049 (3)	0.060 (3)	0.049 (2)	−0.001 (2)	0.0104 (19)	0.006 (2)
C13	0.043 (2)	0.063 (3)	0.071 (3)	−0.006 (2)	0.012 (2)	0.009 (2)
C14	0.037 (2)	0.049 (3)	0.064 (3)	0.0015 (18)	−0.0004 (19)	−0.003 (2)
C15	0.054 (3)	0.059 (3)	0.050 (3)	−0.001 (2)	−0.002 (2)	0.0057 (19)
C16	0.043 (2)	0.056 (3)	0.049 (2)	−0.0029 (19)	0.0094 (18)	0.0049 (19)

### 5-(4-Chlorophenyl)-3-(4-methoxyphenyl)-1,2-thiazole Geometric parameters (Å, º)

**Table:**

S1—N1	1.621 (5)	C9—H9	0.9300
S1—C3	1.711 (4)	C11—C16	1.378 (6)
Cl1—C14	1.754 (4)	C11—C12	1.401 (6)
C4—C5	1.380 (5)	C16—C15	1.394 (6)
C4—C9	1.401 (5)	C16—H16	0.9300
C4—C3	1.461 (5)	C6—H6	0.9300
C1—N1	1.330 (5)	C14—C13	1.365 (7)
C1—C2	1.415 (6)	C14—C15	1.370 (6)
C1—C11	1.472 (6)	C15—H15	0.9300
C7—O1	1.363 (5)	C2—H2	0.9300
C7—C6	1.381 (6)	C12—C13	1.386 (6)
C7—C8	1.405 (6)	C12—H12	0.9300
C8—C9	1.368 (6)	C13—H13	0.9300
C8—H8	0.9300	C10—O1	1.381 (6)
C5—C6	1.395 (5)	C10—H10A	0.9600
C5—H5	0.9300	C10—H10B	0.9600
C3—C2	1.366 (5)	C10—H10C	0.9600
			
N1—S1—C3	96.9 (2)	C11—C16—H16	119.2
C5—C4—C9	118.0 (4)	C15—C16—H16	119.2
C5—C4—C3	121.0 (3)	C7—C6—C5	119.5 (4)
C9—C4—C3	120.9 (4)	C7—C6—H6	120.3
N1—C1—C2	114.6 (4)	C5—C6—H6	120.3
N1—C1—C11	120.3 (4)	C13—C14—C15	121.7 (4)
C2—C1—C11	125.1 (3)	C13—C14—Cl1	120.2 (4)
C1—N1—S1	109.5 (3)	C15—C14—Cl1	118.1 (4)
O1—C7—C6	125.6 (4)	C14—C15—C16	118.7 (4)
O1—C7—C8	115.1 (4)	C14—C15—H15	120.6
C6—C7—C8	119.4 (4)	C16—C15—H15	120.6
C9—C8—C7	120.3 (4)	C3—C2—C1	112.6 (3)
C9—C8—H8	119.9	C3—C2—H2	123.7
C7—C8—H8	119.9	C1—C2—H2	123.7
C4—C5—C6	121.7 (4)	C13—C12—C11	121.1 (4)
C4—C5—H5	119.1	C13—C12—H12	119.4
C6—C5—H5	119.1	C11—C12—H12	119.4
C2—C3—C4	130.2 (4)	C14—C13—C12	119.1 (4)
C2—C3—S1	106.4 (3)	C14—C13—H13	120.4
C4—C3—S1	123.3 (3)	C12—C13—H13	120.4
C8—C9—C4	121.1 (4)	O1—C10—H10A	109.5
C8—C9—H9	119.4	O1—C10—H10B	109.5
C4—C9—H9	119.4	H10A—C10—H10B	109.5
C16—C11—C12	117.7 (4)	O1—C10—H10C	109.5
C16—C11—C1	122.2 (4)	H10A—C10—H10C	109.5
C12—C11—C1	120.1 (4)	H10B—C10—H10C	109.5
C11—C16—C15	121.6 (4)	C7—O1—C10	118.2 (4)
			
C2—C1—N1—S1	−0.9 (5)	C12—C11—C16—C15	1.3 (6)
C11—C1—N1—S1	177.7 (3)	C1—C11—C16—C15	−178.4 (4)
C3—S1—N1—C1	0.0 (3)	O1—C7—C6—C5	179.2 (4)
O1—C7—C8—C9	−179.1 (4)	C8—C7—C6—C5	−0.9 (6)
C6—C7—C8—C9	0.9 (6)	C4—C5—C6—C7	0.1 (6)
C9—C4—C5—C6	0.6 (6)	C13—C14—C15—C16	0.3 (7)
C3—C4—C5—C6	−178.2 (4)	Cl1—C14—C15—C16	−179.3 (4)
C5—C4—C3—C2	19.7 (6)	C11—C16—C15—C14	−1.1 (7)
C9—C4—C3—C2	−159.1 (4)	C4—C3—C2—C1	175.8 (4)
C5—C4—C3—S1	−163.3 (3)	S1—C3—C2—C1	−1.6 (4)
C9—C4—C3—S1	17.9 (5)	N1—C1—C2—C3	1.7 (5)
N1—S1—C3—C2	1.0 (3)	C11—C1—C2—C3	−176.8 (4)
N1—S1—C3—C4	−176.6 (3)	C16—C11—C12—C13	−0.6 (6)
C7—C8—C9—C4	−0.2 (6)	C1—C11—C12—C13	179.0 (4)
C5—C4—C9—C8	−0.6 (6)	C15—C14—C13—C12	0.3 (8)
C3—C4—C9—C8	178.3 (4)	Cl1—C14—C13—C12	179.9 (4)
N1—C1—C11—C16	167.0 (4)	C11—C12—C13—C14	−0.2 (7)
C2—C1—C11—C16	−14.5 (6)	C6—C7—O1—C10	−1.2 (6)
N1—C1—C11—C12	−12.6 (6)	C8—C7—O1—C10	178.9 (5)
C2—C1—C11—C12	165.9 (4)		

### 5-(4-Chlorophenyl)-3-(4-methoxyphenyl)-1,2-thiazole Hydrogen-bond geometry (Å, º)

**Table:**

*D*—H···*A*	*D*—H	H···*A*	*D*···*A*	*D*—H···*A*
C9—H9···S1	0.93	2.77	3.155 (4)	106
C12—H12···N1	0.93	2.55	2.857 (6)	100
